# Implementing a sandbox approach in health technology assessment: benefits and recommendations

**DOI:** 10.1017/S0266462324000412

**Published:** 2024-11-04

**Authors:** Jamie Elvidge, Nick Crabb, Diana Delnoij, Saskia Knies, Douglas Lundin, François Houÿez, Juha Röning, Junfeng Wang, Li Jiu, Alastair Bennett, Yingying Zhang, Dalia Dawoud

**Affiliations:** 1 Science Evidence and Analytics Directorate, National Institute for Health and Care Excellence, UK; 2 Erasmus School of Health Policy and Management, Erasmus University, The Netherlands; 3 National Health Care Institute, Diemen, Rotterdam, The Netherlands; 4 Department of the General Director, The Dental and Pharmaceutical Benefits Agency, Stockholm, Sweden; 5 Treatment Information and Access Department, EURORDIS, Paris, France; 6 Biomimetics and Intelligent Systems Group, University of Oulu, Oulu, Finland; 7 Division of Pharmacoepidemiology and Clinical Pharmacology, Utrecht University, Utrecht, The Netherlands; 8 Centre for Health Economics, University of York, York, UK; 9 Faculty of Pharmacy, Cairo University, Cairo, Egypt

**Keywords:** health technology assessment, HTA, innovation, sandbox, policy sandbox

## Abstract

**Objectives:**

The sandbox approach, developed in the financial technologies sector, creates an environment to collaboratively develop and test innovative new products, methods and regulatory approaches, separated from business as usual. It has been used in health care to encourage innovation in response to emerging challenges, but, until recently, has not been used in health technology assessment (HTA). This article summarizes our learnings from using the sandbox approach to address three challenges facing HTA organizations and to identify implications for the use of this approach in HTA.

**Methods:**

We identified three challenging contemporary HTA-related topics to explore in a sandbox environment, away from the pressures and interests of “live” assessments. We convened a pool of 120 stakeholders and experts to participate in various sandbox activities and ultimately co-develop solutions to help HTA organizations respond to the identified challenges.

**Results:**

Important general learnings about the potential benefits and implementation of a sandbox approach in HTA were identified. Consequently, we developed recommendations to guide its use, including how to implement an HTA sandbox in an effective way and the types of challenges for which it may be best suited.

**Conclusions:**

For many HTA organizations, it is difficult to carefully consider emerging challenges and innovate their processes due to risks associated with decision errors and resource limitations. The sandbox approach could reduce these barriers. The potential benefits of addressing HTA challenges in a collaborative “safe space” are considerable.

## Introduction

Health technology assessment (HTA) organizations sit at the juncture between science and policymaking. They are typically expected to evaluate the body of evidence to respond to a specific policy or decision inquiry about one or more health technologies (e.g., new medicines). They are often expected to consider many different types of evidence, each with its own evolving scientific best practice, and are increasingly assessing highly innovative and disruptive health technologies that stretch traditional HTA processes, such as one-time curative treatments. These pose important challenges for HTA organizations and may increase the risk of HTA decision error, such as delaying access to cost-effective innovative treatments or recommending the use of a treatment that is in fact not cost-effective. The risks associated with a resulting misallocation of limited healthcare resources, to the HTA organization’s reputation, the payer’s finances, wider stakeholders, and most importantly, patients, are potentially significant.

Despite the scientific challenges that HTA organizations face, it can be difficult for them to address these challenges by innovating HTA processes. They are typically constrained by available financial and personnel resources being committed to conducting timely assessments. In addition, as public sector organizations, good governance dictates that they act in a consistent and predictable way, which lends itself to the risk-averse use of established, familiar methods. One relatively new mechanism to facilitate innovation is the “sandbox” approach. Pioneered in the financial technologies sector (1) (where stringent regulation to protect against financial loss can be a barrier to product innovation), the sandbox provides a “safe space” to innovate and co-develop solutions in a collaborative way, isolated from live, business-as-usual activities and associated risks. Such safe spaces might help HTA organizations to develop and test solutions to high-priority challenges in a collaborative way, without affecting ongoing assessments and decisions. We sought to pilot the sandbox in the field of HTA and examine its potential benefits and practical considerations.

This study has been supported by Next-Generation Health Technology Assessment (HTx), which is a Horizon 2020 project supported by the European Union, lasting for 5 yr from January 2019. Its main aim is to create a framework for the next generation of HTA to support patient-centered, societally oriented, and real-time decision-making on access to and reimbursement for health technologies throughout Europe.

## Methods

### Systematic literature review

To establish a picture of how the sandbox approach has been used in health care, a systematic literature review was previously conducted to identify examples. The review is detailed in a previous publication (2). Thirteen applications of the sandbox approach in health care were identified, with two distinct primary purposes. *Testing environment* sandboxes have created spaces to develop and test innovative products and processes, isolated from usual activities and risks. *Regulatory* sandboxes have sought to ensure regulation has not acted as a barrier to innovation, and may also be used in an anticipatory way to shape regulation and standards for an emerging field (e.g., digital health (3)) or challenge (e.g., a humanitarian crisis (4)). However, no applications of the approach in the field of HTA were identified.

### Application of an HTA sandbox

To start to understand the potential benefits and practical considerations of using a sandbox approach to address HTA challenges, we sought to implement what is, to our knowledge, the first *HTA sandbox.* Our HTA sandbox was primarily deployed to develop innovative potential solutions to those challenges, rather than also testing them. While no universally agreed definition of the sandbox approach exists, our sandbox would follow common principles identified from previous applications (1;2;5;6):

1. Create a “safe space” environment that is isolated from ongoing business-as-usual activities, in which participants can develop or test new approaches.
2. Create a neutral environment that fosters collaborative working with a broad range of relevant stakeholders.
3. Establish a clear scope to ensure participants understand the issue being addressed by the sandbox and the intended output.

From 2021 to 2023, members of the HTx project consortium identified three distinct topics which pose, or are expected to pose, challenges for the HTA community. We convened a multidisciplinary pool of relevant stakeholders to participate in various sandbox activities designed to understand each challenge and co-develop the solutions. In this paper, we briefly summarize this process and the resulting outputs, but predominantly focus on the general implications for the use of the sandbox approach in HTA. Detailed topic-specific methods and findings are available elsewhere (7) and others will soon be.

### Identification of sandbox topics

*Assessment of therapeutics and diagnostics for COVID-19.* Governments acted urgently to address the rapid onset of Coronavirus disease (COVID-19), largely without input from HTA organizations (7), though some did find innovative ways to quickly contribute to the wider response (8). It was clear that the HTA community would eventually need to review and assess interventions for COVID-19 that entered practice in the urgent response phase and thereafter, and those assessments were likely to be complex. This presented an opportunity to pilot the sandbox approach to address an upcoming HTA challenge.

*Shared decision making.* HTA organizations can increasingly expect to be presented with interventions that are data-driven tools to inform clinical decision-making, such as digital health technologies (9). As methods to interrogate large and varied data sets improve, such digital tools are likely to be able to provide results that are more relevant for patients at the individual or subgroup level, guiding more personalized treatment decisions. They may pose particular assessment, implementation, and ethical challenges. To support HTA organizations in assessing such interventions, a resource to help developers consider their acceptability and generate HTA-ready evidence from an early stage would be valuable.

*Outcomes-based payment methods.* Regulators are increasingly granting marketing authorization for medicines based on early or uncertain evidence of clinical effectiveness, which leads to uncertainty in subsequent HTA decision-making. Payers are finding ways to mitigate their financial risk through innovative risk-sharing agreements, such as outcomes-based reimbursement agreements (OBAs) that involve further data collection (10). Advanced therapy medicinal products (ATMPs), characterized by high up-front costs and uncertain long-term outcomes, are often difficult technologies to assess (11) and OBAs may help to de-risk or otherwise manage some of those challenges. However, they can be challenging to implement (12), and a strategy to guide their appropriate use would be valuable (13).

### Identification of multidisciplinary experts

**Figure 1.**
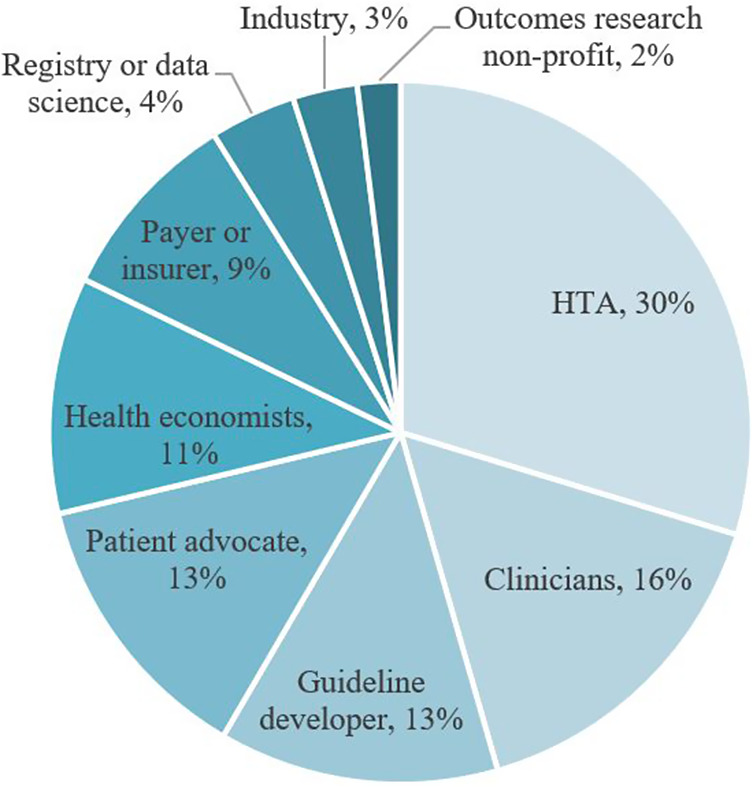
Composition of HTx sandbox expert pool.

Having identified three topics on which to focus the HTA sandbox, we convened an expert pool of 120 multidisciplinary expert stakeholders, predominantly from across Europe (see Figure 1). Participants were identified through networks of members of the HTx project and search for key opinion leaders in each topic. We invited the most appropriate members to contribute to the sandbox activities for each topic, totalling fifty-four for the COVID-19 topic, thirty-one for shared decision making, and thirty-five for OBAs. All sandbox engagement activities were conducted virtually. We provided information about what the activities would involve, how results would be analysed, and that no personal or sensitive data would be collected.

## Results

### Summary of sandbox activities

For the topic of assessing therapeutics and diagnostics for COVID-19, sandbox activities involved extensive work, including reviews, online surveys and virtual workshops, to fully understand the key challenges that HTA organizations would face when evaluating interventions for COVID-19. This was followed by the co-development of an HTA best-practice guide to support HTA organizations when approaching those challenges (7). The shared decision-making topic involved a targeted literature review to understand barriers and facilitators to shared decision-making tools; online surveys about existing tools, including relapsing–remitting multiple sclerosis treatment tool previously developed by the HTx project (14); and a virtual workshop to facilitate the co-development of recommendations to increase their acceptability and implementability. Finally, for the topic of using OBAs to reimburse ATMPs, sandbox activities focused on a series of multidisciplinary virtual workshops to co-develop a process and recommendations to guide when and how to consider and implement an OBA.

### HTA sandbox outputs

The HTA sandbox activities culminated in four main outputs. First, a best practice guide for use by HTA organizations was developed, to guide their assessments of interventions for COVID-19 in light of the known challenges they pose. Second was a series of recommendations for use by the developers of data-driven tools to inform shared treatment decisions, to improve their acceptability to users and the likelihood of implementation into practice. Third, we developed a systematic process to guide payers and HTA organizations in considering the appropriateness of deploying an OBA with data collection to reimburse ATMPs. Lastly, these outputs and the activities that led to them provided important general learnings regarding the utility and implementation of a sandbox approach to resolving challenging issues for HTA. The authors convened online in October 2023 to review and discuss those insights and form recommendations about the effective use and implementation of the sandbox approach in the field of HTA.

## Discussion

To our knowledge, the work summarized here represents the first applications of the sandbox approach in the field of HTA. Through our experience of applying the sandbox approach to three distinct challenging topics, we have identified some characteristics of challenges that might be effectively addressed this way; namely, where there is likely to be a technical solution for consensus to form around, and where no precedent exists. We have also developed generalizable recommendations for future applications of the approach to address other issues, which complement the earlier recommendations by Leckenby et al. (2). These are:

### a) HTA is most suited to using a sandbox in an anticipatory way

Using the sandbox approach in an anticipatory way can align well with horizon-scanning efforts to identify challenging health technologies, methods or policy developments that HTA organizations expect to face, and where there is no previous experience for them to refer to. To proactively prepare for such challenges, an anticipatory sandbox would provide space for HTA organizations to reflect on their routine practices and experiment with solutions to change them, or develop new processes altogether so that delays to evaluation and access are minimized when an identified challenge arrives. Such horizon-scanning efforts should be sufficiently forward-looking to identify emerging issues at an early stage.

### b) A sandbox could reduce duplication of efforts by HTA organizations

Many HTA organizations face common challenges (11), which they might approach unilaterally, duplicating efforts and potentially reaching different solutions that decrease certainty among stakeholders. Centralizing HTA sandbox activities to some extent would reduce such duplication and increase consistency between HTA organizations. Sandbox activities themselves require resources; for example, to identify relevant expert stakeholders and organize engagement activities, and horizon scanning to identify upcoming challenges. Those resources could be drawn from multiple HTA organizations. For example, a European-level consortium project could be established specifically to provide HTA sandbox infrastructure as a service, to pilot and refine novel methods in an environment that is isolated from “live” assessments. The recently established Horizon Europe project Support Utilisation of Sustainable and TAilored INnovative methods for HTA (SUSTAIN-HTA) represents a timely example of this (15).

### c) An HTA sandbox is most useful for challenges that have a technical solution and to support iterative technology development

Convening multiple HTA organizations and multidisciplinary stakeholders will mean participants in a given sandbox activity may represent a range of different perspectives, local needs, and policy contexts. It might be difficult for such a group to identify common solutions to normative challenges relating to, for example, value judgements and ethical issues. Instead, an HTA sandbox may be most useful for addressing challenges that are expected to have an objective technical solution for stakeholders to agree upon, particularly where there is no existing best practice to follow, or for inputting into innovative technology development to ensure HTA methods are evolving to accommodate the challenges posed by new technologies.

### d) More contentious topics may be suited to national or regional HTA sandboxes

Although challenges with an objective technical solution may be the most fruitful for an HTA sandbox, no topic should be considered entirely inappropriate for the sandbox way of working. More contentious issues (including ever-present “big ticket” HTA topics such as discounting and perspective) might never be explored without a safe, semi-academic environment separate from business as usual. They may be better suited to sandbox activities undertaken at the national level, such as the recently established National Institute for Health and Care Excellence HTA Innovation Laboratory (HTA Lab) (16), or at the regional level between HTA organizations that are closely aligned (e.g., Beneluxa).

### e) Clearly communicate the scope, objectives and safe space

There will be times when the different stakeholders participating in an HTA sandbox have different interests in its outcome. The “owner” of an HTA sandbox should think carefully about which stakeholders are needed to hold a successful sandbox for a given challenge and consider all contributions in the context of the likely motivation of the contributor. To mitigate such challenges, it is crucial to position an HTA sandbox in isolation from normal work processes, including concurrent assessments, where conflicts of interest would be at their highest. This should be communicated through clear definitions of the scope and objectives of the sandbox, explaining that it is a semi-academic pursuit with the goal of examining a challenging topic in a collaborative way to co-develop solutions that may not exist yet.

### f) Alignment with the generic innovation framework (IHTAM)

**Figure 2.**
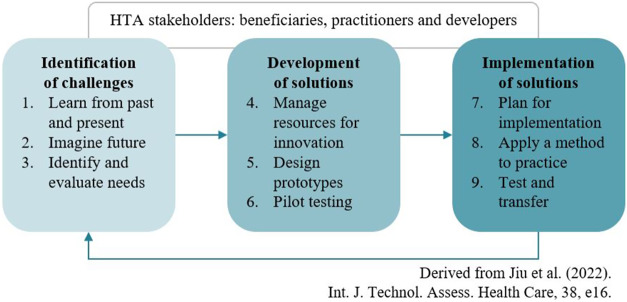
Simplified overview of the Innovation in HTA Methods framework (17).

The Innovation in HTA Methods (IHTAM) framework has been developed by the HTx project (17) to guide innovation in HTA methods. It sets out three generic phases that HTA organizations can follow to systematically identify a need, develop an innovative method or approach, and implement it (see Figure 2). We identified intuitive alignment between the sandbox approach and the IHTAM framework; in particular, sandbox activities are likely to be used in the framework’s “identification” (of challenges) and “development” (of innovative solutions) phases. The “implementation” phase is more likely to be addressed by individual, national HTA organizations considering the merits of the sandbox’s outputs. When we applied the generic framework to our three HTx sandbox topics, all could be positioned in one or more of the framework’s sub-phases, and the authors found it a useful way to contextualize the sandbox activities. Therefore, we advise that the IHTAM framework and the sandbox approach are complementary tools to support innovation in HTA.

Using a sandbox framework that is to some degree centralized (for example, being led by a European research consortium) may allow individually resource-constrained HTA organizations to avoid duplicating their efforts and share knowledge to resolve common technical challenges. Bigger issues in HTA, and topics relating to ethics and value judgements, could be examined using the sandbox approach but may be better suited to doing so on a more local scale.

Since the initiation of this work, at least one HTA organization has now formally adopted the sandbox approach to address challenging and disruptive health technologies in an anticipatory way (16). This complements other initiatives that focus on the open, collaborative exchange of ideas and solutions (e.g., the HTAi Global Policy Forum (18)). Using an explicit sandbox approach establishes clear up-front objectives, boundaries, and expectations for all participants. If more HTA organizations formally adopt the approach, they should carefully consider their ability to act on the findings from sandbox activities, perhaps by dedicating sandbox resources to focus on the implementation of its outputs. Otherwise, it could become a purely academic and inefficient use of public HTA resources.

**Figure 3.**
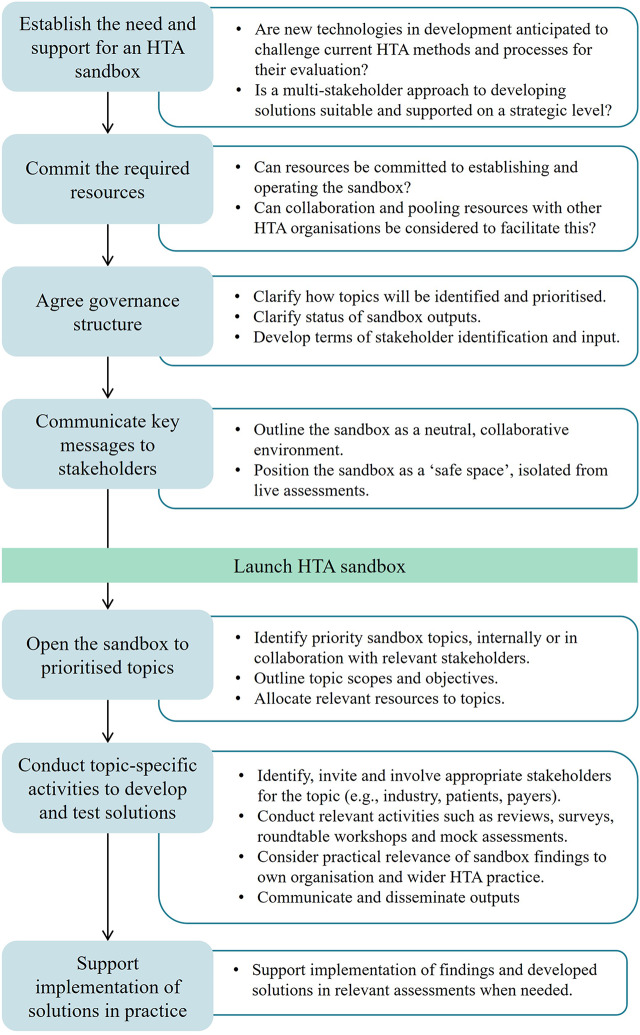
Step-by-step process for implementing an HTA sandbox.

Based upon the generalizable findings listed above, a step-by-step process has been developed to outline some of the key stages involved in the effective implementation of a sandbox in HTA (Figure 3). This may provide useful information for HTA organizations that are considering if and how they might use the sandbox approach.

## Limitations

Our application of a sandbox in HTA had some limitations that may affect the generalizability of our recommendations for implementing the approach. Most prominently, we were not able to demonstrate the sandbox as a *testing environment* for a novel product or process. This was theoretically possible for the shared decision-making topic, where the prototype multiple sclerosis tool would ideally have been iteratively refined during sandbox activities. However, further development of the tool was not possible at that time. Instead, we used the approach to generate more generic recommendations to support the future development of such tools. The COVID-19 and OBA topics were examples of using an HTA sandbox in an *anticipatory* way, and developing HTA process solutions to known or upcoming challenges.

Like any research involving the collaboration of external participants, our HTA sandbox can only reflect the views and expertise of the stakeholders who agreed to participate. A different pool of stakeholders may have led to different levels of quality of engagement, outputs and recommendations.

## Conclusions

HTA organizations sit at the juncture of science and policy-making. In this context, establishing a sandbox and implementing it as a way of working can help them to innovate and address known challenges, such as the emergence of new methods, diseases, and disruptive technologies. The approach involves embedding a collaborative mentality among relevant experts and stakeholders and conducting activities that primarily seek to *co-develop the right technology or method.* Sandbox activities and outputs must be isolated from ongoing assessments, which are naturally subject to competing conflicts of interest and pose risks to all stakeholders of reaching an incorrect conclusion. They are likely to be most useful when undertaken in collaboration with the developers of disruptive technologies or innovative methods at the very early stages of research and development, to ensure the framework used to evaluate or implement them is fit for purpose and minimise any problems during evaluation. Similar to the financial technologies industry that founded the sandbox concept and the regulatory sandboxes developed and used by regulators, HTA is a field where risks are potentially very costly. These represent barriers to developing and testing solutions that are different to the status quo, which an HTA sandbox could remove. The potential benefits to HTA organizations of anticipating challenges and working to resolve them in a forward-looking way are considerable, but dedicated resources are required to implement an effective sandbox. Where appropriate, organizations should seek to collaborate on sandbox activities.
